# The Use of Naltrexone to Successfully Treat Severe Hailey-Hailey Disease

**DOI:** 10.7759/cureus.110281

**Published:** 2026-06-05

**Authors:** Fatemeh Mehrabian, Emily Vaughan, Maaz Abid, Nirav Gandhi, Rand Hawari

**Affiliations:** 1 General Internal Medicine, University Hospitals of Derby and Burton NHS Foundation Trust, Derby, GBR; 2 Dermatology, University Hospitals of Derby and Burton NHS Foundation Trust, Derby, GBR; 3 Pathology, University Hospitals of Derby and Burton NHS Foundation Trust, Derby, GBR; 4 Histopathology, University Hospitals of Derby and Burton NHS Foundation Trust, Derby, GBR

**Keywords:** antibiotics therapy, genodermatosis, low-dose naltrexone, stable pharmacological remission, systemic steroids

## Abstract

A 65-year-old gentleman presented with a three-year history of burning, itching, and splitting of the scrotal skin. He had previously been treated with topical corticosteroids, emollients, antifungals, and multiple short and long courses of antibiotics, with limited response. A biopsy was performed, which confirmed a diagnosis of Hailey-Hailey disease (HHD). HHD is an autosomal dominant genodermatosis characterised by vesicles that form eroded, erythematous plaques with painful rhagades in flexural areas.

Oral naltrexone has been used to treat alcohol use disorder and opioid use disorder for many years. Naltrexone is known to block opioid receptors and indirectly antagonize toll-like receptor 4 in the microglial cells of the central nervous system and peripheral macrophages. This potentially reduces inflammation due to a decrease in the production of substance P, tumour necrosis factor, interleukin-6 (IL-6), and other pro-inflammatory cytokines.

In 2022, acitretin was commenced and uptitrated to 25 mg once daily. Unfortunately, his condition deteriorated, with extensive erosions affecting the groin and scrotum, and the patient also developed side effects from the medication. A surgical opinion was sought, but surgery was not considered suitable. Courses of prednisolone did not help. Other immunosuppressive agents, such as methotrexate, cyclosporine, and azathioprine, have been described in the literature with varying efficacy.

After two years, the pain from his erosions had reduced his ability to walk. Naltrexone 2.5 mg was commenced and subsequently uptitrated to 5 mg. Six weeks later, the patient had an 80% improvement in his condition. The patient experienced no side effects from the treatment and required no blood monitoring.

This gentleman with severe HHD predominantly affecting the scrotum was commenced on oral naltrexone. Naltrexone is postulated to work due to its analgesic and anti-inflammatory effects. It is a well-tolerated treatment for severe HHD that has drastically improved both our patient's skin and his quality of life.

## Introduction

Hailey-Hailey disease (HHD), also known as familial benign chronic pemphigus, is a rare inherited acantholytic dermatosis first described by the Hailey brothers in 1939 [1]. It is usually inherited in an autosomal dominant pattern and is caused by pathogenic variants in the ATP2C1 gene, which encodes the secretory pathway calcium/manganese ATPase 1 (hSPCA1), a calcium pump involved in intracellular calcium regulation and keratinocyte adhesion [2,3]. Disruption of calcium homeostasis leads to impaired desmosomal adhesion between keratinocytes, producing the characteristic suprabasal acantholysis seen histologically, often described as a “dilapidated brick wall” appearance [4].

Clinically, HHD typically follows a chronic relapsing-remitting course, with recurrent painful erosions, vesicles, fissuring, maceration, and secondary infection, most commonly affecting intertriginous areas such as the axillae, groin, neck, inframammary folds, and genital region [4,5]. Flares may be triggered or worsened by heat, sweating, friction, trauma, and infection, making long-term disease control challenging [5]. Although HHD is not life-threatening, it can substantially impair quality of life because of pain, pruritus, malodour, recurrent infection, functional discomfort, and psychological distress [6].

Management remains difficult, as no curative treatment is currently available and responses to conventional therapies are often incomplete or temporary [5,7]. Reported treatment options include topical corticosteroids, topical calcineurin inhibitors, antiseptics, topical and systemic antibiotics, antifungals, retinoids, immunosuppressive agents, botulinum toxin, laser therapy, dermabrasion, and surgical approaches, but no single regimen is consistently effective for all patients [7]. Low-dose naltrexone (LDN) has emerged as a potential therapeutic option for refractory HHD, with several case reports and small case series describing meaningful clinical improvement, including in patients with disease resistant to multiple previous therapies [8-11]. Its proposed benefits may relate to modulation of cutaneous opioid receptors, anti-inflammatory effects, and possible improvement in keratinocyte adhesion, although the precise mechanism in HHD remains uncertain [8,9].

We report the case of a 65-year-old man with severe, treatment-resistant HHD who achieved sustained clinical improvement with oral LDN, maintaining disease control for more than two years without reported adverse effects. This case was presented at the 2025 BAD Annual Meeting on July 2, 2025. This case adds to the growing literature supporting LDN as a promising, low-cost, and well-tolerated therapeutic option in selected patients with refractory HHD.

## Case presentation

A 65-year-old gentleman presented with a three-year history of burning, itching, fissuring, and splitting of the scrotal skin, associated with recurrent skin infections. He had previously received several topical treatments, including emollients, moderate-potency topical corticosteroids, and miconazole/hydrocortisone cream, with limited benefit. He had also received multiple short courses of antibiotics, including ciprofloxacin and flucloxacillin. His past medical history included atrial fibrillation, transient ischaemic attack, and osteopenia. Initial investigations, including full blood count, viral hepatitis, HIV, liver function tests, urea and electrolytes, folate, vitamin B12, thyroid function tests, and HbA1c, were unremarkable (Tables 1-2).

**Table 1:** Microbiology results. HBV: Hepatitis B virus; HCV: Hepatitis C virus.

Test/sample	Result
HIV	Negative
HBV	Negative
HCV	Negative
Skin swab	Heavy growth of *Staphylococcus aureus*

**Table 2:** Biochemistry tests. Na: Sodium; K: Potassium; eGFR: Estimated glomerular filtration rate; ALP: Alkaline phosphatase; ALT: Alanine aminotransferase; INR: International normalized ratio; APTT: Activated partial thromboplastin time; Hb: Hemoglobin; WCC: White cell count; HbA1c: Glycated hemoglobin; TSH: Thyroid-stimulating hormone; PSA: Prostate-specific antigen; HDL: High-density lipoprotein.

Test	Value	Units	Normal range
Na	141	mmol/L	133-146
K	4.2	mmol/L	3.5-5.3
Urea	5.2	mmol/L	2.5-7.8
Creatinine	82	µmol/L	59-104
eGFR	83	mL/min/1.73 m²	
Bilirubin	10	µmol/L	10-21
ALP	108	U/L	40-129
ALT	13	U/L	0-40
Total protein	68	g/L	60-80
Albumin	35	g/L	35-50
INR	1.32	ratio	0.9-1.1
APTT	30.5	seconds	20.2-29.1
Hb	137	g/L	130-170
WCC	9.7	×10⁹/L	4.0-10.0
Platelets	308	×10⁹/L	150-450
CRP	1.1	mg/L	0-5
Adjusted calcium	2.32	mmol/L	2.20-2.60
Ferritin	163	µg/L	30-400
HbA1c	31	mmol/mol	<48
TSH	2.56	mIU/L	0.5-4.5
Magnesium	0.81	mmol/L	0.7-1.4
Phosphate	0.9	mmol/L	0.8-1.5
PSA	2.44	µg/L	0.1-6.5
Vitamin D	81	nmol/L	50-144
Folate	3.8	µg/L	3.8-26.8
Vitamin B12	310	ng/L	197-771
HDL cholesterol	1.07	mmol/L	0.94-1.48
Non-HDL cholesterol	4.03	mmol/L	
Triglycerides	2.21	mmol/L	0.5-1.7

**Figure 1:**
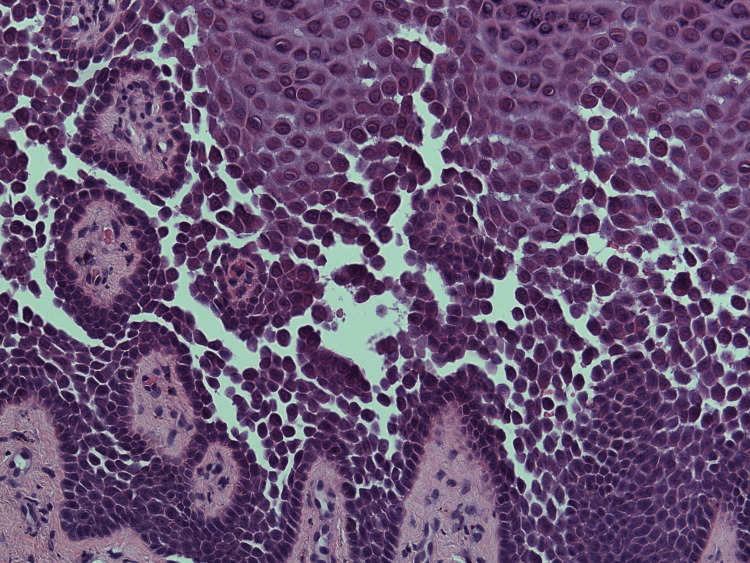
Histopathological image with H&E staining, original magnification ×200.

**Figure 2:**
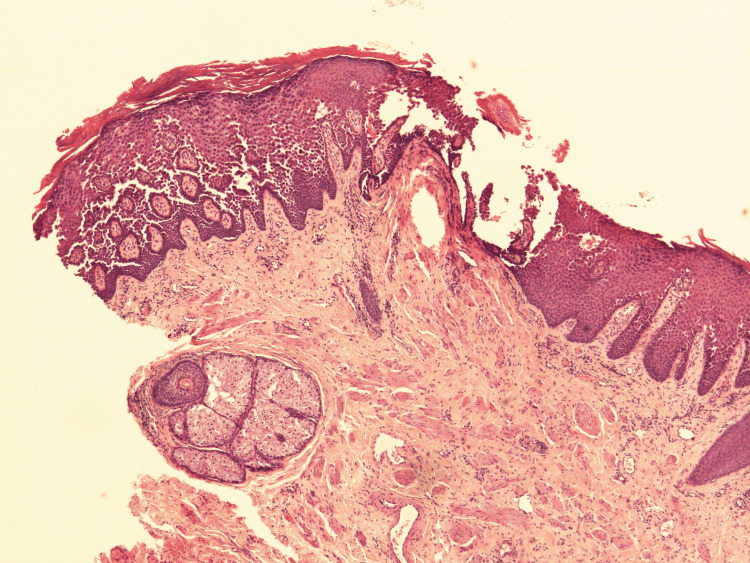
Histopathological image with H&E staining, original magnification ×40.

A 4-mm punch biopsy was performed. Histology showed hyperkeratosis, patchy parakeratosis, prominent epidermal acanthosis, and widespread acantholysis involving multiple levels of the squamous epithelium, producing a “dilapidated brick wall” appearance. Occasional dyskeratotic cells were also noted. The overall clinicopathological findings were consistent with HHD (Figures 1-2). Histologically, acantholysis throughout the epidermis is a recognised hallmark of HHD [4].

The patient was commenced on oral lymecycline 408 mg once daily alongside potent topical corticosteroids. At follow-up, he reported persistent symptoms with frequent flare-ups. Lesions had extended to involve the trunk and neck, in addition to the scrotum and groin. His topical treatment was escalated to a very potent topical corticosteroid, and lymecycline was switched to doxycycline. He was also assessed for suitability for acitretin. In 2022, acitretin was commenced at 25 mg twice weekly and subsequently titrated to 25 mg once daily. However, the treatment was later discontinued at the patient’s request because of intolerable stinging scrotal pain with no improvement in symptoms.

His condition subsequently deteriorated, with extensive erosions, fissuring, and oozing affecting the groin and scrotum. A surgical opinion was sought from both urology and plastic surgery; however, no appropriate surgical intervention was identified.

After two years of persistent disease, pain from the erosions had significantly reduced his ability to walk and socialise, with a profound impact on his quality of life. He was commenced on oral prednisolone in an attempt to achieve disease control through systemic immunosuppression, but the response was disappointing, with minimal improvement. Other systemic immunosuppressive agents, including methotrexate, ciclosporin, and azathioprine, have been reported in HHD with variable efficacy, but their role in routine clinical practice remains limited [7]. Interventional approaches, including ablative techniques, photodynamic therapy, ultraviolet B therapy, and botulinum toxin, have also been described, although these were not considered appropriate for this patient at that stage [7].

A literature review identified several reports describing clinical improvement in refractory HHD with oral low-dose naltrexone (LDN) [8-13]. Among emerging treatments for HHD, LDN is an attractive option because it is relatively inexpensive, generally well tolerated, and has been associated with meaningful improvement in several case reports and case series [8-13]. However, the available evidence remains limited, and further controlled studies are needed before its role in treatment algorithms can be clearly defined. Naltrexone is an opioid receptor antagonist traditionally used in the management of alcohol and opioid use disorders [14]. At low doses, its proposed effects in inflammatory and acantholytic dermatoses may involve transient opioid receptor blockade, modulation of endogenous opioid signalling, and immunomodulatory effects through inflammatory cytokine pathways, although the precise mechanism in HHD remains uncertain [14-16].

**Figure 3:**
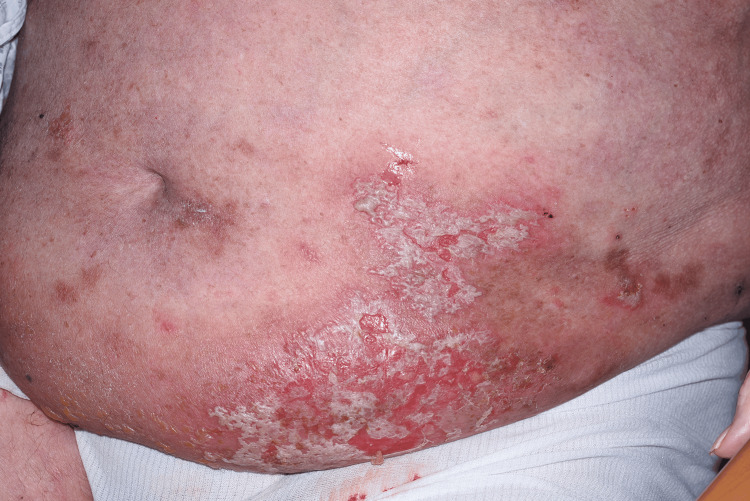
Before treatment with low-dose naltrexone.

**Figure 4:**
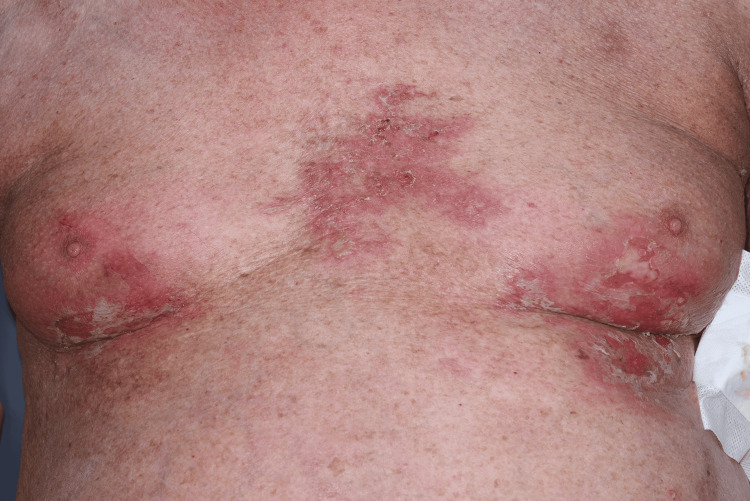
Before treatment with low-dose naltrexone.

**Figure 5:**
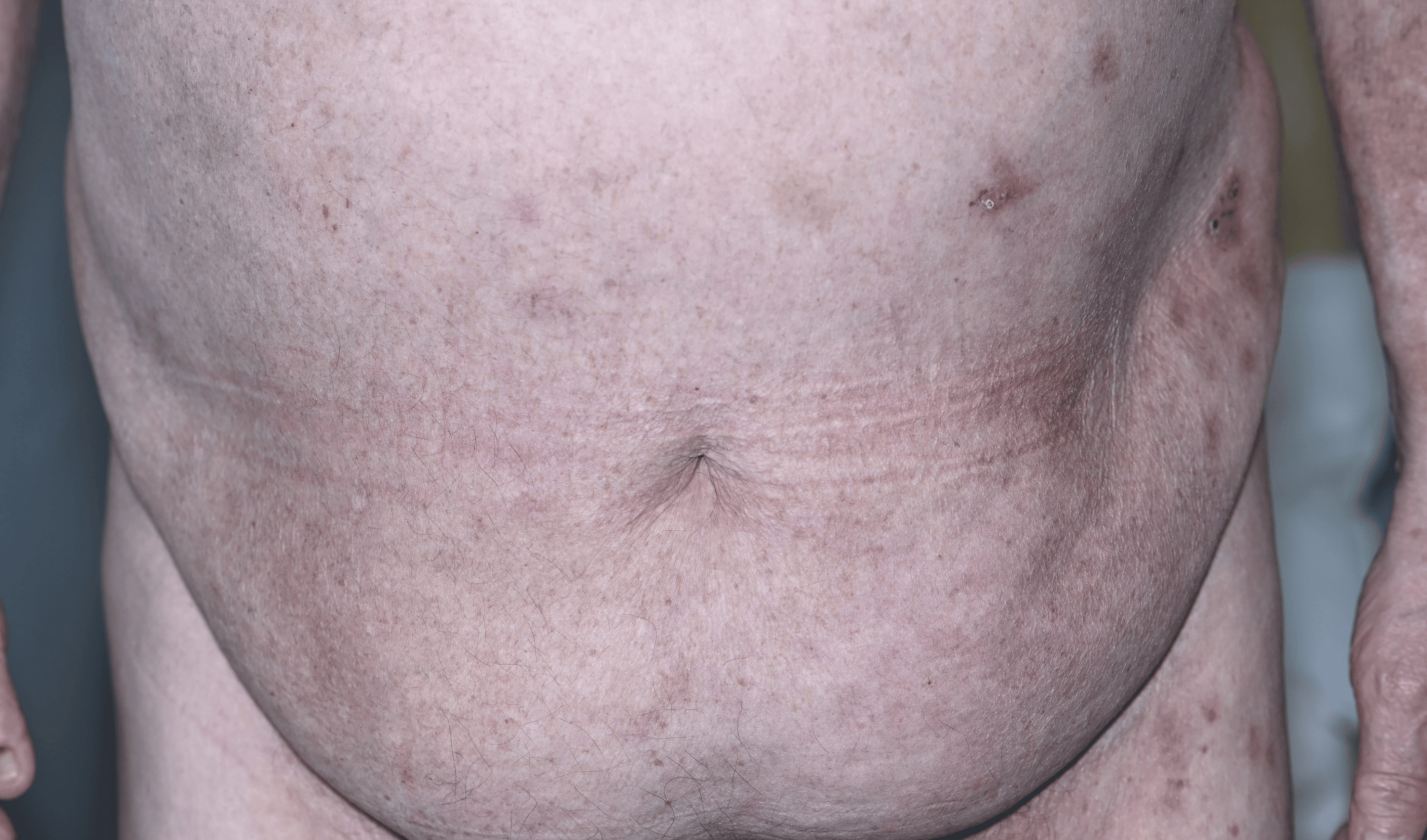
After treatment with low-dose naltrexone.

**Figure 6:**
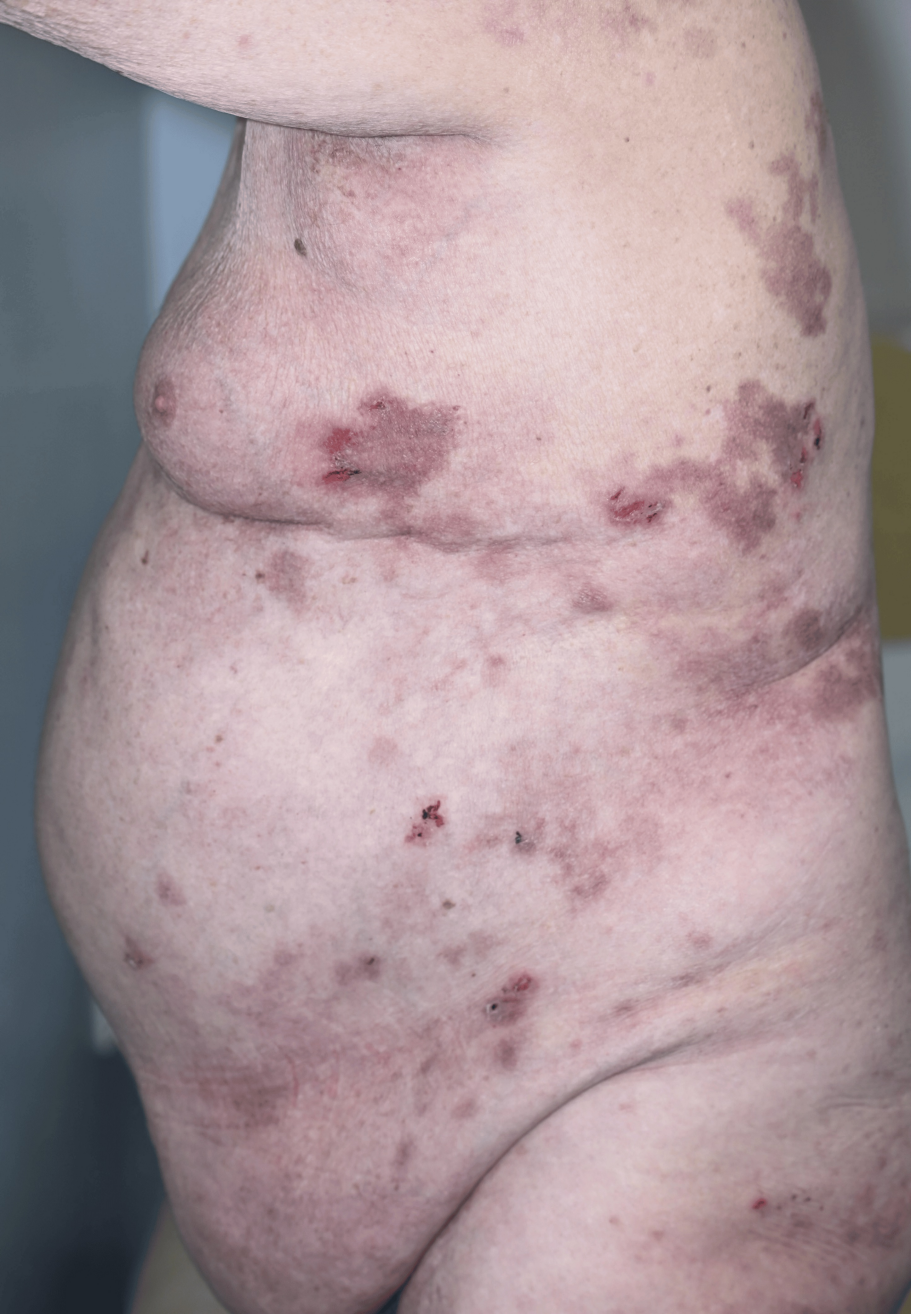
After treatment with low-dose naltrexone.

Following approval through the local medicines governance process, the patient was commenced on low-dose oral naltrexone 2.5 mg once daily. This was titrated to 5 mg once daily after six weeks. At the six-week follow-up, the patient reported marked symptomatic improvement (Figures 3-6). He initially experienced occasional flare-ups, which were thought to be infection-driven and were associated with positive wound swab cultures.

The patient was also counselled regarding avoidance of recognised exacerbating factors for HHD, including heat, sweating, friction, and excessive sun exposure. Practical advice included wearing loose, breathable clothing, keeping affected areas cool and dry, avoiding prolonged heat exposure where possible, and using regular photoprotection, including broad-spectrum sunscreen and physical sun avoidance measures during periods of high ultraviolet exposure.

Over the following two years, the patient experienced only one significant flare-up, which occurred after excessive sun exposure during a holiday. His disease has otherwise remained well controlled on naltrexone 5 mg once daily, with no reported adverse effects. He remains under dermatology follow-up on a three-monthly basis.

## Discussion

Hailey-Hailey disease (HHD) is a chronic relapsing acantholytic dermatosis that can be difficult to manage, particularly when it affects intertriginous or genital sites. Although HHD is not usually life-threatening, painful erosions, fissuring, recurrent infection, malodour, and functional limitation can cause substantial impairment in quality of life [6,7]. This was evident in our patient, whose scrotal and groin involvement caused persistent pain, recurrent infection, reduced mobility, and significant psychosocial distress despite multiple treatment attempts.

Management of HHD remains challenging because there is no curative therapy, and responses to conventional treatments are often incomplete or temporary [7]. Standard approaches include trigger avoidance, topical corticosteroids, topical calcineurin inhibitors, antiseptics, topical or systemic antimicrobials, retinoids, and systemic immunosuppressive agents [7]. Procedural treatments, including botulinum toxin, laser therapy, photodynamic therapy, dermabrasion, and surgery, have also been described, although suitability depends on disease distribution, severity, patient preference, and local availability [7]. In our case, the patient had failed or not tolerated several treatments, including topical corticosteroids, oral antibiotics, acitretin, and systemic corticosteroids. Surgical options were explored but were not considered appropriate.

Low-dose naltrexone (LDN) has emerged as a promising treatment option for refractory HHD, although current evidence is limited mainly to case reports and small case series [8-13,17-20]. Ibrahim O et al. reported improvement in patients with familial benign chronic pemphigus treated with LDN and suggested that it may offer a low-cost, low-risk alternative or adjunctive therapy [8]. Similar clinical improvement has been described by Albers L et al., Kollman N and Bass J, Alajmi A et al., Campbell V et al., and Riquelme-Mc LC et al. [9-13,19,20]. However, responses are not uniform. Cao S et al. reported variable outcomes among patients treated with naltrexone, highlighting that LDN is unlikely to be universally effective and that predictors of response remain unclear [17].

Our case adds to this growing literature by demonstrating marked and sustained improvement with LDN in a patient with severe, functionally limiting, treatment-resistant HHD. After titration from 2.5 mg once daily to 5 mg once daily, the patient reported approximately 80% subjective improvement at six weeks, particularly in pain, burning, fissuring, and irritation. Clinical review demonstrated marked improvement, with reduced erythema, maceration, erosions, and splitting of the affected skin, supported by comparison with baseline photographs. Although validated measures such as the Dermatology Life Quality Index (DLQI) or Physician Global Assessment (PGA) were not recorded prospectively, the combination of patient-reported improvement, objective clinical findings, photographic evidence (Figures 3-6), and sustained response during follow-up supports a clinically meaningful therapeutic benefit. More importantly, disease control was maintained over two years, with only one significant flare following excessive sun exposure and no reported adverse effects. This prolonged follow-up is clinically relevant, as HHD typically follows a relapsing-remitting course, and many published reports describe shorter follow-up periods.

The mechanism by which LDN may improve HHD remains uncertain. Naltrexone is an opioid receptor antagonist traditionally used at higher doses in the treatment of alcohol and opioid use disorders [14,15]. At lower doses, it is thought to produce transient opioid receptor blockade, which may modulate endogenous opioid signalling and inflammatory pathways [14]. Naltrexone may also have anti-inflammatory effects through antagonism of toll-like receptor 4 (TLR4) signalling, with reduced production of inflammatory mediators such as tumour necrosis factor-alpha (TNF-α), interleukin-6 (IL-6), and substance P [14,16]. These effects may be relevant in HHD, where inflammation, pain, pruritus, infection, and impaired keratinocyte adhesion contribute to disease activity. However, the precise mechanism in HHD remains theoretical and requires further study.

Trigger avoidance remains an important adjunctive strategy in the management of HHD. Heat, sweating, friction, and sun exposure are recognised exacerbating factors [5] and may contribute to recurrent flares, particularly in flexural or occluded sites. In addition to pharmacological therapy, patients should be advised about practical measures such as minimising friction, wearing loose clothing, maintaining careful skin hygiene, keeping affected areas cool and dry, avoiding excessive heat and sweating, and using photoprotection where ultraviolet exposure appears to trigger disease activity. In this case, the patient reported flares following sun exposure, and counselling regarding heat avoidance and photoprotection was therefore included as part of adjunctive management.

The main strength of this case is the sustained clinical response over a two-year period in a patient with refractory disease who had limited remaining treatment options. LDN was well tolerated, inexpensive, and did not require intensive monitoring, making it a practical option in this clinical context. However, this is a single case report and cannot establish treatment efficacy. HHD has a naturally fluctuating course, and improvement may also have been influenced by infection control, trigger avoidance, or concurrent topical measures. In addition, no validated disease severity score or quality-of-life measure was used, so response was assessed clinically and through patient-reported improvement.

In conclusion, this case supports the growing evidence that LDN may be a useful treatment option for selected patients with refractory HHD. Our patient achieved marked improvement with naltrexone 5 mg once daily and maintained disease control for more than two years without reported adverse effects. Although larger prospective studies are needed before LDN can be incorporated confidently into treatment algorithms, it appears to be a promising, accessible, and well-tolerated option in difficult-to-treat HHD.

## Conclusions

In summary, this case describes a gentleman with severe, treatment-resistant HHD who achieved marked and sustained improvement after commencing oral low-dose naltrexone. Treatment was well tolerated and was associated with significant improvement in skin symptoms and quality of life, with disease control maintained for more than two years. Although further studies are needed to clarify its efficacy, optimal dosing, and place within treatment pathways, low-dose naltrexone appears to be a promising, low-cost, and accessible option for selected patients with refractory HHD.
